# *Mesobuthus eupeus* venom induced injury in the colorectal carcinoma cell line (HT29) through altering the mitochondria membrane stability

**DOI:** 10.22038/ijbms.2020.40884.9659

**Published:** 2020-06

**Authors:** Massood Valizade, Atefeh Raesi Vanani, Mohsen Rezaei, Laya Sadat Khorsandi, Leila Zeidooni, Masoud Mahdavinia

**Affiliations:** 1 Cell & Molecular Research Center, Ahvaz Jundishapur University of Medical Sciences, Ahvaz, Iran; 2 Toxicology Research Center, Ahvaz Jundishapur University of Medical Sciences, Ahvaz, Iran; 3 Department of Toxicology, School of Pharmacy, Ahvaz Jundishapur University of Medical Sciences, Ahvaz, Iran; 4 Department of Toxicology, Faculty of Medical Sciences, Tarbiat Modares University, Tehran, Iran; 5 Department of Anatomical Sciences, Faculty of Medicine, Ahvaz Jundishapur University of Medical Sciences, Ahvaz, Iran

**Keywords:** AFM, Apoptosis, HT-29 cancer cell, Mesobuthus eupeus venom, Mitochondrial membrane – potential, ROS

## Abstract

**Objective(s):**

The purpose of this study was to investigate cytotoxicity and membrane toxicity effects induced by *Mesobuthus eupeus* venom (MEV) on the HT-29 cell line.

**Materials and Methods:**

To determine the *in vitro* cytotoxicity via MTT assays, HT-29 (as cancer cell line) and Hek-293T (as normal cell) were treated through different concentrations of MEV, and cytotoxicity effects were then measured through assessment of mitochondrial membrane potential (ΔΨm), reactive oxygen species (ROS) generation, and apoptosis induction. The colony formation assay was performed to measure the antiproliferative effect of MEV on HT-29 cells. Nuclei alterations were also observed during apoptosis following DAPI staining. Besides, atomic force microscopy (AFM) was used to detect alterations in morphology and ultrastructure of the cells at a nanoscale level.

**Results:**

According to MTT and clonogenic assays, MEV caused a significant decrease in cell viability and proliferation of HT-29 cells while it did not have any impact on normal cells and the IC50 value was found to be 10 µg/ml. Induction of apoptosis was also confirmed by flowcytometric analysis in HT-29 cells. Moreover, the results indicated that MEV had led to a suppression of proliferation and induction of apoptosis through increased ROS and depolarization of mitochondria. Furthermore, AFM imaging demonstrated apoptosis cell death after being treated with MEV in HT-29 cells.

**Conclusion:**

This study showed that MEV had an antiproliferative effect on HT-29 cells by inducing apoptosis through the mitochondria signaling pathway. These findings suggested that MEV could be used as a promising natural remedy for cancer treatment.

## Introduction

Cancer is known as one of the leading causes of death around the world. Colorectal cancer (CRC) has been recognized as the third most common tumor in men and the second in women in Western countries, and it has been also considered as the fourth most common cancer-related cause of mortality across the world (1, 2). Radiotherapy, chemotherapy, and surgery have been introduced as key cancer therapies. However, these treatments can have their own severe systemic side effects (3). New potential therapies for cancer have been routinely investigated in preclinical studies and discovering new drugs and agents against cancer, especially those derived from natural products, has been on an increase. 

Nowadays, scorpion venom is deemed as an interesting natural source for cancer therapy (4). Several studies have been similarly carried out in order to identify whether mechanisms of venoms can contribute to finding novel treatments and developing medicines for different diseases (5). The scorpion venom contains salts, nucleotides, biogenic amines, and enzymes, which represent them as good candidates for drug design in the pharmaceutical industry (4, 6, 7). 

Mucroporin-M1 and Kn2-7 are respectively anti-hepatitis B virus and anti-HIV-1 medications that are obtained from scorpion venom. As well, the effects of peptides and drugs against hepatitis C virus and herpes simplex virus type 1 are being investigated (8, 9). The results of a study revealed that scorpion venom had reduced the growth of cancer cells and it was also used to treat human breast and prostate cancer cell lines by Omran (10, 11). MEV is also known as an endemic species from the Middle East belonging to the Buthidae family (12) and there is no scientific evidence for MEV use against cancer cells.

It should be noted that two main mechanisms of cell death are apoptosis and necrosis (13). Apoptosis refers to a physiological cell death that causes the removal of damaging cells in normal conditions. Therefore, any disorder in apoptosis leads to autoimmune disorders or cancer. Two identified pathways of apoptosis include intrinsic and extrinsic pathways and mitochondria are the main regulators of the intrinsic pathway. Under physiological conditions, mitochondria can also have a membrane potential, called ΔΨm. In this respect, a great decrease in ΔΨm is a primary event in apoptosis signaling pathways (14). ROS at high concentrations can correspondingly cause irreversible oxidative damage and initiate death of cancer cells through apoptosis, necrosis, or autophagy (15). 

As a nanobiotechnological tool, AFM is used to study morphological cellular membrane surface utilizing a high-magnification electronic microscope, which provides high-resolution images to study normal tissue growth. The cellular membrane also plays a vital role in cell biology including adhesion between cells, cellular structure protection, and cell viability. *In vitro* studies via AFM are also used to detect the type of cell death (16).

The purpose of this study was to investigate the anticancer effect of MEV on the human CRC cell line and also shed light on the mechanisms of cell death induced by MEV. Furthermore, this study aimed to elucidate AFM for visualizing and verifying cell response to MEV.

## Materials and Methods

***Chemicals***

Fetal bovine serums (FBS), Dulbecco’s Modified Eagle Medium (DMEM), Trypan blue, and penicillin-streptomycin were obtained from Gibco BRL (Gaithersburg, MD, USA). As well, 3-(4,5-Dimethylthiazol-2-yl)-2,5-diphenyltetrazolium bromide (MTT), DAPI, dimethyl sulphoxide (DMSO), 2, 7-dichlorofluorescin diacetate (DCFH-DA), phosphate buffer saline (PBS), and trypsin were obtained from Sigma Chemicals (Darmstadt, Germany). In addition, HT-29 (human colon cancer cells) and Hek-293T (human embryonic kidney cells) were acquired from the Iranian Biological Resource Center (IBRC, Tehran, Iran).

***Scorpion venom collection***

Scorpions were collected in Khuzestan Province, in southwestern Iran, and were then identified by Razi Reference Scorpion Laboratory of Ahvaz. Afterwards, MEV was taken via mild electrical simulation (20 V, 500 mA) on a monthly manner and it was solubilized in sterile double distilled water. After centrifuging at 8000 g for 15 min at 4 ^°^C, the supernatant was immediately lyophilized and stored at -20^ °^C for later uses (17). The raw venom was also dissolved in DMEM and protein quantity was consequently assessed through Bradford protein assay (18).

***Cell culture***

 In this part, HT-29 and Hek-293T cells were grown in DMEM medium with 10% FBS, penicillin G (100 U/mL), and streptomycin (100 U/ml), incubated in a CO2 incubator (37 ^°^C, 5% CO_2_, humidified atmosphere), the cells were seeded at a concentration of 1×10^6^ in 25 ml flasks, and then viability of the cells was determined using Trypan blue staining. It should be noted that the medium was exchanged three times a week (19).

***Cell viability***

 The cytotoxicity effect of MEV was determined via MTT assay. To this end, cells (1×10^6^) were seeded in 96-well plates and incubated overnight for recovery and cell adhesion in a humidified atmosphere of 5 % (v/v) CO_2_ at 37 ^°^C. Following the incubation process, the cells were treated with different concentrations of MEV (0, 3, 10, 30, and 90 µg/ml), which were then dissolved in DMEM and again incubated at 37 °C for 24, 48, and 72 hr. Cells with culture medium and without MEV were also used as untreated growth controls. After the incubation process, the MTT solution was added to each well (5 mg/ml) and it was then incubated for 4 hr. MTT was then reduced by mitochondrial dehydrogenases in viable cells and produced purple-colored formazan. The crystals of formazan were correspondingly dissolved in DMSO and computed by measuring the absorbance at 570 nm by an ELISA plate reader. Absorbance from untreated cells was considered as 100 % of growth and it was used for viability calculation. The effect of MEV on the viability of human cell lines was consequently expressed as %viability, using the formula: %viability = A_570 _of treated cells/A_570 _of control cells×100 %. The IC_50_ value of cancer cells was also determined. It should be noted that the experiments were performed five times to determine cell viability (MTT reduction) (20).

***ROS measurement***

ROS measurement was conducted using H2DCFDA according to the manufacturer’s instructions. In brief, each 24-well plate was seeded with 2×10^4^ cells per well and allowed to adhere overnight. Moreover, 10 μg/ml MEV was added for the specified period. H2O2 was also used as a positive control. Following drug treatment, the media were removed and the cells were loaded with 5 mM H2DCFDA diluted in clear media for 30 min at 37 °C. The cells grown in multi-wall plates needed to be washed twice with PBS to remove the medium. Next, 0.5 ml of measurement buffer containing 2 mM CM-H2DCFDA was added. Immediately after addition, fluorescence measurement in kinetic mode at 485 nm excitation and 520 nm emission wavelengths was started (21).

***Flow cytometric analysis***

Flow cytometry with Annexin V-FITC/7AAD and PI was done to distinguish between live/ apoptotic/necrotic cells after treatment of HT-29 and Hek-293T ones with MEV (IC_50_: 10 µg/ml) (Vermes *et al*. 1995). In brief, 1.5_10^6^ cells were seeded and treated with 10 µg/ml of MEV in DMEM for 24 hr. Following drug exposure, the cells were harvested, washed with ice-cold phosphate-buffered saline (PBS), and stained with an Annexin V-FITC/7AAD kit (Beckman Coulter, Villepinte, France) for 15 min in darkness. In all, 450 ml of binding buffer was added to terminate the reaction. The samples were kept on ice before being subjected to flow cytometric analysis. Stained samples were also analyzed on a flow cytometer (Dako Cytomation Cyan LX, Dako Corp., Carpinteria, CA, USA) and 10000 events were collected. The data were then plotted using Summit V4.3 Build 2445 software (Dako Colorado Inc., Fort Collins, CO, USA) (22). One flask of cells that was not exposed to any inducer, as a negative control, and another flask of cells exposed to anisomycyne, as a positive control, were also stained according to the kit protocol (23).

***Mitochondrial membrane potential (*** **ΔΨm** ***) determination***

Briefly, the cells were seeded in 24-well plates at a density of 1×10^6^ cells/well and then incubated overnight. Afterward, the medium was replaced and the cells were also exposed to 10 µg/ml of MEV and incubated for 24 hr. The harvested cells were correspondingly incubated in 200 nM of MTG (Mitotracker green) for 30 min at 37 °C in darkness. Then, cell suspensions were placed in a melting ice bath and analyzed via a flow cytometer (23). One flask of cells that was not exposed to any inducer, as a negative control, and another flask of cells exposed to anisomycyne (2 µg/ml, 2 hr), as a positive control, were also stained according to the kit protocol.

***Clonogenicity assay***

In order to investigate the MEV antiproliferative effect on HT-29 cells, colony formation assay was carried out. For this purpose, 500 cells were seeded into 6-well culture dishes. After 24 hr, the cells were exposed to MEV in a serum-free medium at appropriate times. Then, the cells were washed and incubated with complete medium for 10 days. The cells were also washed with 1×PBS and fixed with 4% paraformaldehyde at room temperature for 20–30 min. After washing them with 1×PBS again, they were stained for 10 min in 0.1% crystal violet/PBS, and the colonies were counted. All experiments were performed in triplicate. The colonies which had ≥50 cells per colony were also counted. The data were represented as the number of colonies formed per 500 cells plated and then converted into percentages (24).

***DAPI staining***

Five hundred cells were seeded into 6-well culture dishes. In this respect, HT-29 cells were grown on coverslips after treatment with MEV for 24 hr. Nuclei changes of apoptosis were then assayed by DAPI staining. Following rinse with PBS, the cells were fixed with cold methanol/acetone for 5 min at room temperature. They were then rinsed with PBS three times and incubated with DAPI solution (2 mg/ml in PBS) for 10 min in darkness at room temperature. To remove the unbound DAPI, the cells were washed with PBS. Finally, nuclear morphology was imaged through a fluorescence microscope (Olympus, Japan) (25).

***Single cell AFM measurement***

The HT-29 cells were seeded on the slide and treated with MEV (10 µg/ml) for 24 hr at 37 ^°^C in 5% CO_2_. Paraformaldehyde 4% was also used to fix the cells for 15 min; then rinsed with PBS three times, and dried by air at room temperature. An atomic force microscope (Nano Wizard II, JPK Instruments AG, Berlin, Germany) was also used to acquire topographic images in the intermittent mode. To remove any organic contaminants prior to use, silicon tips (ACTA probe; APPNANO) were used in all AFM measurements and were irradiated with UV in the air for 15 min. The ROC and the height of the tip were <6 nm and 14–16 µm, respectively. Cantilever specifications included spring constant set at 40 N/m, length of 125 µm, width of 35 µm, thickness of 4.5 µm, and frequency set at 280 kHz. The samples were then put on the XY AFM scanning station, and >5 cells were measured. In order to remove the background noise at low frequency in the scanning direction, the obtained images were only processed with the onboard software (JPK Image Processing Software, version 3.3) to eliminate low-frequency background noise in the scanning direction.

***Statistical analysis***

All statistical analyses were performed using GraphPad Prism Software (version 5.04). Continuous variables were also expressed as mean±SD and then compared using one-way analysis of variance (ANOVA); followed by Tukey’s *Post hoc* test. Statistical significance was also set at *P*-value<0.05.

## Results

***Effect of MEV on HT29 cell viability***

**Figure 1:**
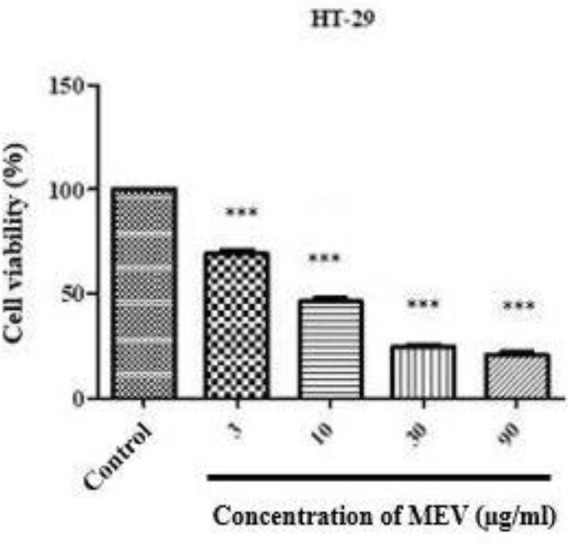
MTT reduction in HT-29 cells after 24 hr exposure with different concentrations of *Mesobuthus eupeus* venom (MEV). All assays were performed in triplicate, and the mean±SD was illustrated. *** *P*-value<0.001. *: different levels of significance compared with the control group

**Figure 2:**
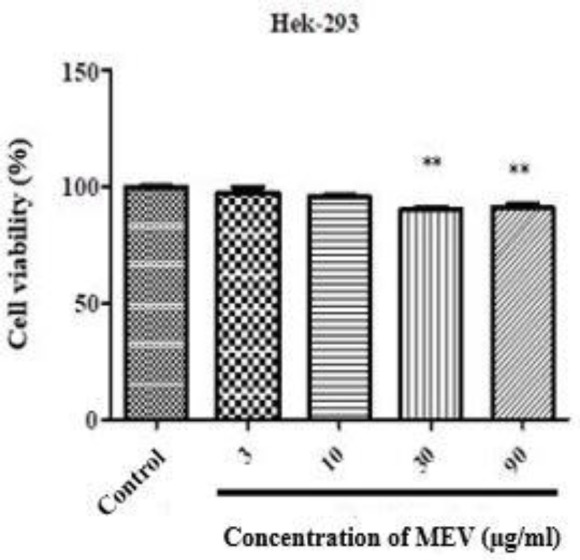
MTT reduction in Hek-293T cells after 24 hr exposure with different concentrations of *Mesobuthus eupeus* venom (MEV). All assays were performed in triplicate, and the mean±SD was illustrated. ** *P*-value<0.01. *: different levels of significance compared with the control group

As shown in Figures 1 and 2, MEV in HT-29 cells led to a decrease in cell viability in a dose-dependent manner. At the doses above 3 µg/ml and higher, cell viability significantly decreased. The IC_50 _parameter was also used to measure the effect of MEV on cell viability in human cell lines. The IC_50 _showed that MEV had exerted a selective viability reduction against HT-29 cells compared with normal ones wherein no effect was observed. In concentrations of 3, 10, 30, and 90 µg/ml, cell viability also decreased, respectively 68.09 % (*P*-value<0.001), 46.13% (*P*-value<0.001), 23.00 % (*P*-value<0.001), and 18.66% (*P*-value<0.001); suggesting that the inhibitory effect of MEV on HT-29 cell viability was dose-dependent. Cell viability had not changed either at concentrations of 3 and 10 µg/ml in Hek-293T cells but decreased at 30 and 90 μg/ml. Therefore, the IC_50 _value of HT-29 cells was measured at 10 µg/ml (46.13%). It should be noted that the significant time was 24 hr and the antiproliferative effect was reduced at the longer time points of 48 and 72 hr.

***ROS measurement***

**Figure 3:**
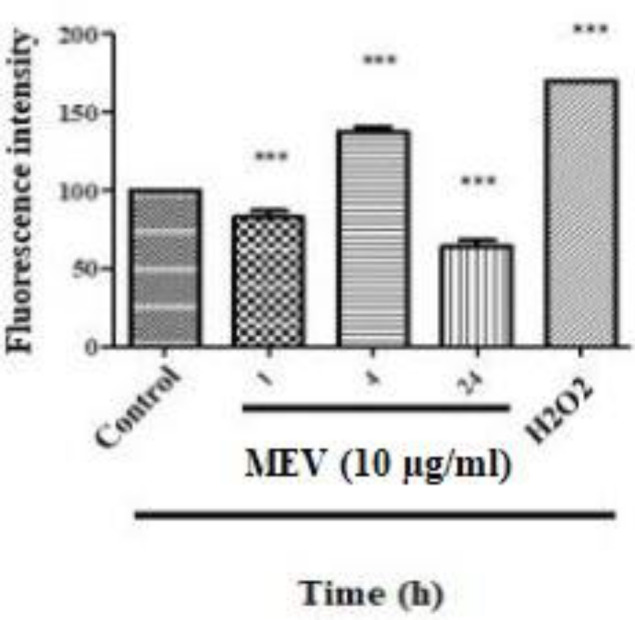
Increased reactive oxygen species (ROS) production after treatment of HT-29 cells with 10 µg/ml of *Mesobuthus eupeus *venom (MEV). All assays were performed in triplicate, and the mean±SD was illustrated. *** *P*-value<0.001. *: different levels of significance compared with negative control cells

The HT-29 cells were loaded with the ROS probe, 2',7'-dichlorodihydrofluorescein diacetate (H2DCFDA), and H_2_O_2_ was included as a positive control. MEV could induce ROS generation in a timely and concentration-dependent manner, as reflected by the increase in fluorescence intensity. As shown in Figure 3, treatment of HT-29 cells with 10 µg/ml of MEV increased the ROS content to a significant (^***^P-value< 0.001) level as compared with the negative control cells. The highest amount of ROS was generated after 4 hr of exposure to 10 µg/ml of MEV.

***Mitochondrial membrane potential (∆*** **Ψm** ***) measurement***

**Figure 4:**
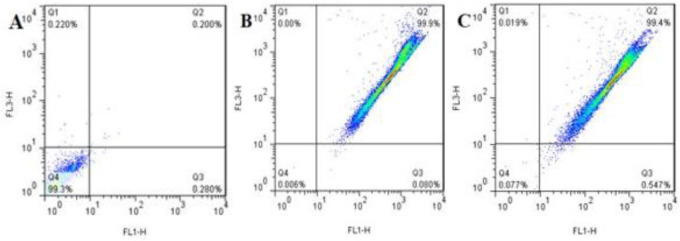
Reduction of ∆Ψm after treatment of HT-29 cells with 10 µg/ml of *Mesobuthus eupeus *venom (MEV): A) negative control, B) treated HT-29 cells with 10 µg/ml of MEV, C) positive control

∆Ψm change could affect cellular health. ROS generation also influenced ∆Ψm and initiated apoptosis. Changes in ∆Ψm were correspondingly measured using MTG (Mitotracker green) dye. As shown in Figure 4, ∆Ψm of the negative control, the treated HT-29 cells with 10 µg/ml of MEV, and the positive control were 0.2%, 99.9%, and 99.4%, respectively. It was also investigated that MEV had changed ∆Ψm and it had decreased significantly at the 10 µg concentrations of MEV compared with those of the negative control.

***Diagnosis of apoptotic cells***

**Figure 5:**
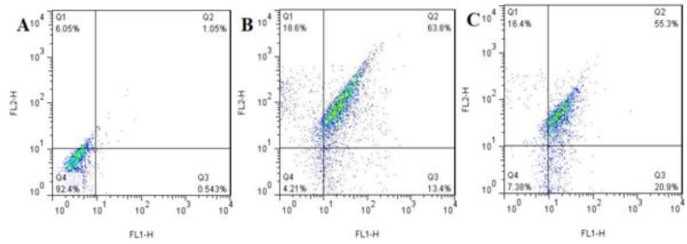
Apoptosis and necrosis ratio of HT-29 cells in negative control cells (A), treated HT-29 cells with 10 µg/ml of *Mesobuthus eupeus *venom (MEV) (B), and positive control cells (C) as measured by flow cytometry with double staining of Annexin V and PI

Flow cytometric data revealed that MEV (10 μg/ml, 24 hr), inducing 13.4% early apoptosis in HT-29 cells, was in the lower right quadrant compared with only 0.543% and 20.9% of negative and positive control cells (2 μg/ml anisomycin, 24 hr), respectively. Moreover, exposure to IC_50_ of MEV for 24 hr induced 63.8% late apoptosis in HT-29 cells that were in the upper right quadrant compared with only 1.05% and 55.3% of negative and positive control cells (IC_50_, 24 hr), respectively. Additionally, the data revealed that MEV (10 μg /ml, 24 hr) had induced 18.6 % necrosis in HT-29 cells that were in the upper left quadrant compared with only 6.05% and 16.4% of negative and positive control cells, respectively (Figure 5).

***Effect of MEV on HT29 cell proliferation***

**Figure 6:**
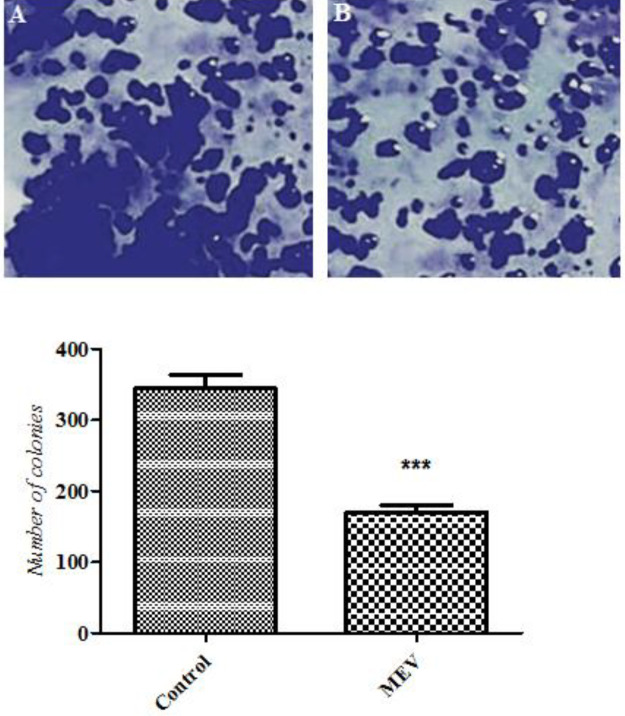
Colony numbers of HT-29 cells before (A) and after (B) treatment with *Mesobuthus eupeus *venom (MEV). The mean±SD were illustrated *** *P*-value<0.001: different levels of significance compared with the control group

Based on the results in MEV treated cells, colony numbers had significantly decreased compared with those in control cells (*P*-value<0.05). The results of the clonogenic assay were also reported in Figure 6.

***DAPI staining***

**Figure 7:**
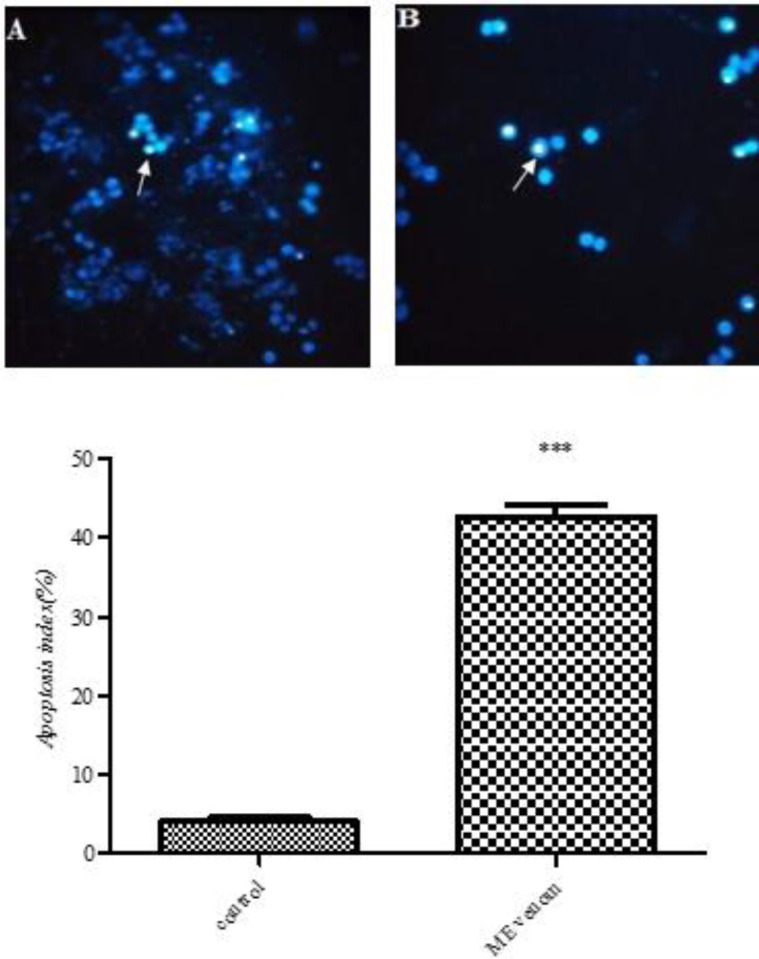
DAPI staining of HT-29 cell nuclei (magnifications: 400), before (A) and after (B) treatment with *Mesobuthus eupeus* venom (MEV). White arrows indicate chromatin condensation. The percentage of apoptotic cells was also shown. The mean±SD were illustrated. *** *P*-value<0.001: different levels of significance compared with the control group

After treatment with MEV for 24 hr, a large number of nuclei with chromatin condensation indicating cell apoptosis were observed in MEV treated cells. The apoptosis index also showed a significant increase in MEV treated cells (Figure 7).

***Changes in cell morphology***

**Figure 8:**
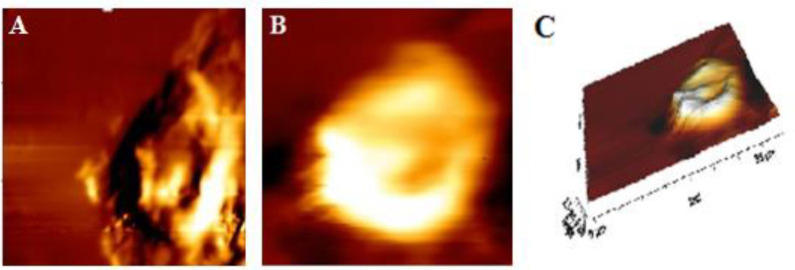
Intermittent mode atomic force microscopy (AFM) image of control HT-29 cells. (A), (B), and (C) are respectively 2D image, phase image, and 3D image

**Figure 9.**
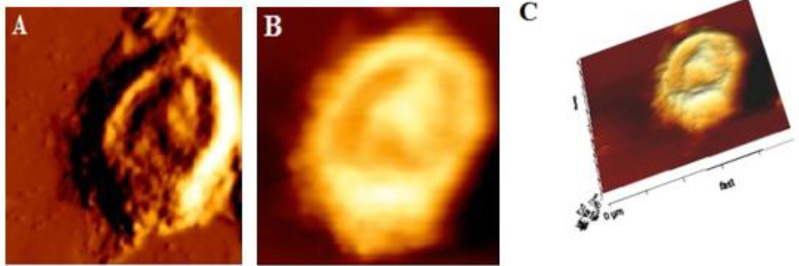
Intermittent mode atomic force microscopy (AFM) image of *Mesobuthus eupeus* venom (MEV) treated HT-29 cells. (A), (B), and (C) are respectively 2D image, phase image, and 3D image

Various changes in surface morphology and ultrastructure of MEV treated cells were observed in the AFM images. The HT-29 cells in the control group were intact (Figure 8A). The cell surface ultrastructure also showed that the cell membrane was relatively smooth and homogeneous and displayed granular morphology with uniform particles (Figures 8B and C). After incubation with 10 mg/ml MEV for 24 hr, cell morphology became irregular (Figure 9A), and then the cell surface became rougher with numerous irregular nanoparticles and some pores (Figures 9B and C), which provided more detailed data on the toxic effect of MEV on cell membranes.

## Discussion

Scorpion venom is known as an attractive natural source for cancer treatment (2, 26). However, the mechanism of MEV against CRC has not yet been identified, and there is no evidence of changes in the morphology of cell membranes. Therefore, the present study was conducted to evaluate the effect of MEV on HT-29 and Hek-293T cells. MTT and clonogenic assays were also used to analyze cell viability and proliferation. Exposing cells to some concentrations of MEV (0, 3,10, 30, and 90 µg/ml), the cell viability in HT-29 cell line decreased to 100%, 68.09%, 46.13%, 23.00%, and 18.66 % after 24 hr, respectively. While significant cytotoxicity, growth inhibition, and selectivity against HT-29 cells were observed as a result of MEV exposure, no effects on Hek-293T cells were identified. In the present study, the results of MTT assay indicated that the IC_50_ of MEV was 10 µg/ml within 24 hr, which had significant effects on HT-29 cells. This could be related to differential expression of cellular targets that are usually recognized by MEV in cancer cells (27). Moreover, the cancerous cells might express various receptors, which are just sensitive to MEV. Considering longer time points of 48 and 72 hr, antiproliferative effects of MEV had reduced, which might be due to MEV denaturation by long-term presence in aqueous solutions.

Although mitochondrial activity is mainly related to metabolism, the activity of this cellular component is not limited to supplying ATP. In other words, cell death is signaled as the second major activity of mitochondria, and mitochondrial ROS also plays a very important role as the initiator of this pathway. One of the major causes of resistance of cancer cells to death is lack of production and release of ROS by mitochondria and this is due to closing of mitochondrial wall pores (MPT) and inefficiency in the mitochondrial pathway of signaling of apoptosis (28). Numerous studies have also shown that oxidative stress can trigger programmed cell death and several antitumor drugs have been introduced to induce apoptosis through raising intracellular ROS and affecting mitochondria in cancer cells (29, 30). In the present study, oxidative stress induced by MEV treatment was verified through increasing total intracellular ROS by DCF-DA staining. Following ROS production, Ψ∆m was expected to decrease. The results also confirmed that MEV had been able to decrease Ψ∆m via inducing ROS production. Following this change, the outer membrane of the mitochondria was permeable, and factors such as ROS and cytochrome-C were released from mitochondria to cell cytosol (31). Via activating cellular cytosol activators, cell apoptosis or necrosis were also expected to be activated. It should be noted that externalization of phosphatidylserine is an early apoptotic occurrence and serves as a binding site for Annexin V-FITC. Counterstaining with PI in this respect can thus provide a distinction between apoptotic and necrotic cells. In this study, Annexin V FITC/PI binding was used to determine the type of cell death through flow cytometry. The results also showed that MEV had induced a higher rate of apoptosis in treated HT-29 cells compared with that in negative control ones.

The ability of a single cell to produce a colony can be confirmed to maintain its capacity for reproduction, or vice versa, to cause damage to chromosomes and apoptosis (32, 33). According to the results of the clonogenic assay, it was confirmed that MEV was able to prevent the formation of colonies by cancer cells. DAPI staining was also used to confirm apoptosis and detect morphology of cell nuclei by binding it to double-stranded DNA in the nuclei. After treatment with MEV, the cell nuclei showed shrinkage and chromatin condensation. According to these data, it could be suggested that MEV could inhibit the proliferation of HT-29 cells via nuclear damage.

**Figure 10:**
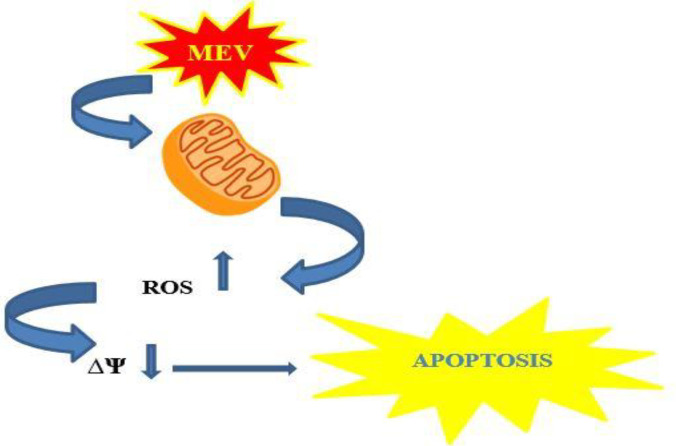
Action mechanism of *Mesobuthus eupeus *venom (MEV)

AFM could provide qualitative and quantitative information about cell membrane morphology. Apoptotic volume decrease (AVD) could also play a vital role in the process of apoptosis cell death, and AFM was considered an appropriate tool to examine cellular apoptosis morphology. The images obtained from AFM showed morphological changes in the surface and the volume of control and treated HT-29 cells with MEV. As a non-destructive photographic instrument, blebbing was ultimately observed and morphological changes were detected in HT-29 cells treated with MEV as a sign of damage to cell core and cytoplasm(34). AFM images also displayed deep pores in cell membranes, which were similar to those of apoptosis (35) after being treated with MEV. Furthermore, there were particles on cell membranes that were bigger than those in the control cells. Some biological events such as changes in the activity of ion channels, disruption of cell structure, or chemical composition changes of outer membrane proteins had been also reported (36, 37). These attributes could thus suggest that susceptibility of cancer cells to MEV treatment was histologically origin-dependent. This was the same feature that might account for the absence of toxicity in normal cells (38, 39). Additional experiments identifying potential pharmacological targets in these cells would also help to characterize the spectrum of action considering the histological source. Some studies have further established the anticancer effect of *Rhopalurus junceus* venom with cytotoxicity effect against a panel of human tumors in epithelial cell lines while it had no impacts on normal cells (2). *Tityus discrepans* venom also exhibited anticancer effects against human breast cancer cell line SKBR3, while no impact was observed in normal monkey kidney cell line MA104 (40). Moreover, MEV venom showed significant cytotoxicity on tumor brain cell line U251-MG while it was not observed in normal cells (41). In addition, venom from *Odontobuthus doriae* had confirmed a cytotoxicity effect against human neuroblastoma cells SH-SY5Y (19). The results of the present study were in line with those of previous research and supported the idea of MEV effect against HT29 cancer cells. Following the changes that MEV had made in increasing ROS and decreasing Ψ∆m, the results of the measurement of apoptosis and necrosis in HT-29 cells showed that MEV was able to kill cancer cells via apoptosis. In general, the mechanism described for the anticancer activity of MEV was the induction of apoptosis via ROS production, mitochondrial depolarization, as well as release of proapoptotic molecules. The possible mechanism of action for *Mesobuthus eupeus* venom was presented in Figure 10. 

## Conclusion

This study sheds light on the anticancer effect induced by MEV against HT-29 cancer cells. It also provided the first scientific evidence demonstrating the effect of MEV on cell viability and proliferation of HT-29 cells. Accordingly, this study found that cytotoxicity and membrane toxicity effects of MEV on HT-29 cells were probably due to the sensitivity of cancer cells. Likewise, it was concluded that MEV could increase ROS, decrease ∆Ψm, and eventually induce apoptosis. Morphological changes were also observed in HT-29 cells as a result of exposure to MEV, leading to apoptosis that was not only an AVD sign, rather it caused damage to the core. Furthermore, this study introduced MEV as a good candidate for future studies on cancer issues.
